# Impairment in Attention Focus During the Posner Cognitive Task in Children With ADHD: An Eye Tracker Study

**DOI:** 10.3389/fped.2020.00484

**Published:** 2020-09-02

**Authors:** Simona Caldani, Frederic Isel, Mathilde Septier, Eric Acquaviva, Richard Delorme, Maria Pia Bucci

**Affiliations:** ^1^UMR 7114 MoDyCo, CNRS-Université Paris Nanterre, Nanterre, France; ^2^EFEE-Centre d'exploration fonctionnelle de l'équilibre chez l'enfant, Robert Debré Hospital, Paris, France; ^3^Child and Adolescent Psychiatry Department, Robert Debré Hospital, Paris, France; ^4^Paris University, Paris, France; ^5^Human and Genetic Functions, Institut Pasteur, Paris, France

**Keywords:** attention, eye-tracking, ventral, dorsal, brain network

## Abstract

Attention is a major cognitive function that allows the individuals to focus selectively on a discrete stimulus while ignoring others. Visual information could be driven endogenously, when the goals or desires are voluntary, or exogenously, in response to salient visual events in the environment. Since subjects with attention deficit hyperactivity disorder (ADHD) show heightened distractibility during activities that require significant attentional engagement, we hypothesized that they may be more severely impaired in their ability to perform endogenous tasks than controls. To elicit endogenous and exogenous shifts of attention, we thus used a modified version of Posner's cueing task. We compared oculomotor performance measured by an eye tracker in a group of 31 children with ADHD (mean age = 9.1 ± 1.3 years) and age-, sex-, and IQ-matched typically developing children. Endogenous and exogenous conditions were explored in three distinct visual sub-conditions (valid, invalid, and neutral). We found that children with ADHD showed longer latency during endogenous conditions compared to TD children in invalid sub-conditions. They also performed more errors than controls, during the endogenous task in neutral sub-conditions and during exogenous task in neutral and invalid sub-conditions. Our study suggests that children with ADHD may allocate their attention resource toward the detection of exogenous targets with a deficit in their ability to perform endogenous task. We suggest also that they have a difficulty in the engagement of the inhibitory control system particularly during voluntary saccade performance. This could result from impaired interactions between the ventral and dorsal attention networks as well as in the frontal eye field, although neuroimaging studies are necessary to validate this hypothesis in the ADHD population.

## Highlights

- Children with ADHD were impaired in their ability to perform endogenous tasks in the invalid sub-condition as compared to controls as they showed longer eye movement latency during a modified Posner's cueing task.
- Longer latencies during endogenous conditions could be explained by altered interactions between the ventral and dorsal attention networks in children with ADHD.
- Elevated number of errors in children with ADHD could be explained by frontal eye field dysfunction.

## Introduction

Attention is a major cognitive function that allows the individuals to focus selectively on a discrete stimulus while ignoring others (1). The biological substrates involved in spatial selection of attention toward internal or external stimuli seem to be distinct (2). The selection of exogenous stimuli is mainly mediated by dorsal frontoparietal regions, including the intraparietal sulcus (3) while the endogenous (re)-orienting toward task-relevant events involves also the ventral frontal and temporo-parietal regions, including the temporo-parietal junction (4, 5).

Attention deficit/hyperactivity disorder (ADHD) is a neurodevelopmental disorder with symptoms of impaired attention, impulsivity, and hyperactivity, which occurs in about 5% of children and 2.5% of adults (6). Despite inattention being a core feature of ADHD, the spatial orientation (endogenous vs. exogenous tasks) of attention in subjects with ADHD remains a question of debate (7, 8).

In experimental research, one can indirectly detect the attention focalization on an exogenous object of interest by the ability of one subject to perform saccadic eye movements to place this object on the fovea (9). Derived from these findings, the neuropsychological Posner task was built to assess the individual's ability to perform an attentional shift by exploring the attentional disengagement from endogenous or exogenous tasks. In the endogenous condition, a central cue (often a central arrow) predicts the location of a target and induces a voluntary shift of attention. In the exogenous condition, a peripheral cue, like peripheral luminance onsets, drives attention shift automatically (10). Interestingly, saccadic eye movements and shifts of attention share similar brain networks, such as the frontal eye field, the lateral intraparietal cortex, and the superior colliculi (11, 12). Impairments in attention perception during endogenous tasks in adult subjects with ADHD are correlated with a right-hemisphere anterior deficit (13), but results were inconsistent among studies (14). In contrast, there are consistent findings (but with a reduced effect size) showing a deficit of attention disengagement in the cognitive treatment of exogenous cues (15, 16). In summary, there is no consistent evidence for specific dysfunction in the voluntary orienting of attention in patients with ADHD.

Moreover, Weinstein et al. (17) measured the pupil size in subjects with ADHD during a Sternberg-type delayed visuospatial working memory task and they pointed out that changes in pupil size during a cognitive task could be useful for a better and complete ADHD diagnosis.

Note that to our knowledge, no studies have been done by examining eye movements in a Posner task in children with ADHD.

The aim of this study was to explore further the performance in the Posner task in children with ADHD in order to observe how they are able to engage or disengage their attention. We hypothesized that children with ADHD may be more severely impaired in their ability to perform endogenous tasks than controls since their attention resources were more allocated to the detection of exogenous targets (9, 18). To answer to this question, we examined the eye movement latency in a Posner task in children with ADHD. Note that latency values could be used to probe the integrity of information processing and the allocation of attentional resources.

## Materials and Methods

### Participants

Thirty children with ADHD (mean age = 9.1 ± 1.3 years) and thirty-one IQ- and age-matched typically developmental (TD) children were recruited at the Child and Adolescent Psychiatry Department, Robert Debré Hospital (Paris, France). All children were evaluated by trained child psychiatrists. The diagnosis of ADHD according to DSM-5 criteria (19) was carried out using the Kiddie-SADS-EP (20). During the interview, the presence of psychiatric comorbidities (for instance, anxiety, obsession, and other mental disorders) was systematically screened for and it represented an exclusion criterion for our study. The severity of ADHD symptoms was estimated by using the ADHD Rating Scale (ADHD-RS) parental report. All children with ADHD were also assessed for their cognitive abilities by using the Wechsler Intelligence Scale for Children (fourth edition), the Beery-Buktenica Developmental Test of Visual-Motor Integration (VMI) (21), and the Motor Assessment Battery for Children (MABC) (22). Note also that all children did not receive any psychotherapeutic treatment or other type of therapeutic intervention.

TD children were recruited from the general population. To be included in our study, controls should have an ADHD-RS total score ≤ 10 (23) and a neurological examination in the normal range. Their cognitive skills were evaluated by using two subtests exploring verbal ability (the similarities test) and performance ability (matrix reasoning test). The visual acuity at near distance (Parinaud test) was measured for all children tested.

The clinical characteristics of children with ADHD and controls are reported in Table 1. One TD child was excluded from the study since he had visual acuity below the normal range after corrections (<20/20).

**Table 1:** Clinical characteristics of children enrolled in the study (after exclusion of one child with typical development for visual acuity below the normal range after corrections).

	**TD**	**ADHD**
	***N* = 30**	***N* = 31**
**Clinical data**		
Age (years)	9.5 ± 2.3	9.1 ± 1.3
Visual acuity	20/20	20/20
**ADHD-RS**		
ADHD-RS total score	4 ± 0.8	38.8 ± 1.8
ADHD-RS inattention subscore	–	19.3 ± 1
ADHD-RS hyperactivity/impulsivity subscore	–	19.5 ± 1.2
**Wechsler scale (WISC-IV) scores**		
Verbal Comprehension subscale	–	99.1 ± 3.3
Perceptual reasoning subscale	–	93.6 ± 3.5
Working memory subscale	–	86.6 ± 3.0
Processing speed subscale	–	90.9 ± 2.7
Similarity test	10.06 ± 0.4	9.9 ± 0.6
Matrix reasoning test	10.14 ± 0.5	9.7 ± 0.5

*TD, typically developing; ADHD, attention deficit hyperactivity disorder, N, number; ADHD-RS, attention deficit hyperactivity disorder rating scale; WISC-IV, Wechsler Intelligence Scale for Children*.

The investigation adhered to the principles of the Declaration of Helsinki and was approved by the local ethic committee (Comité de Protection des Personnes CPP, Ile de France). After the nature of the procedure had been explained, a written informed consent was obtained from all participants and their parents.

### Visual Condition Experiment

To elicit endogenous and exogenous shifts of attention, we used a modified version of Posner's cueing task (14). In the endogenous condition, centrally presented arrows were used to induce voluntary shifts of attention. In the exogenous condition, peripheral luminance onsets were used to draw attention automatically. The background display consisted of two empty white boxes, with a white fixation cross in the center of the screen, which were presented continuously during the entire run.

**Figure 1:**
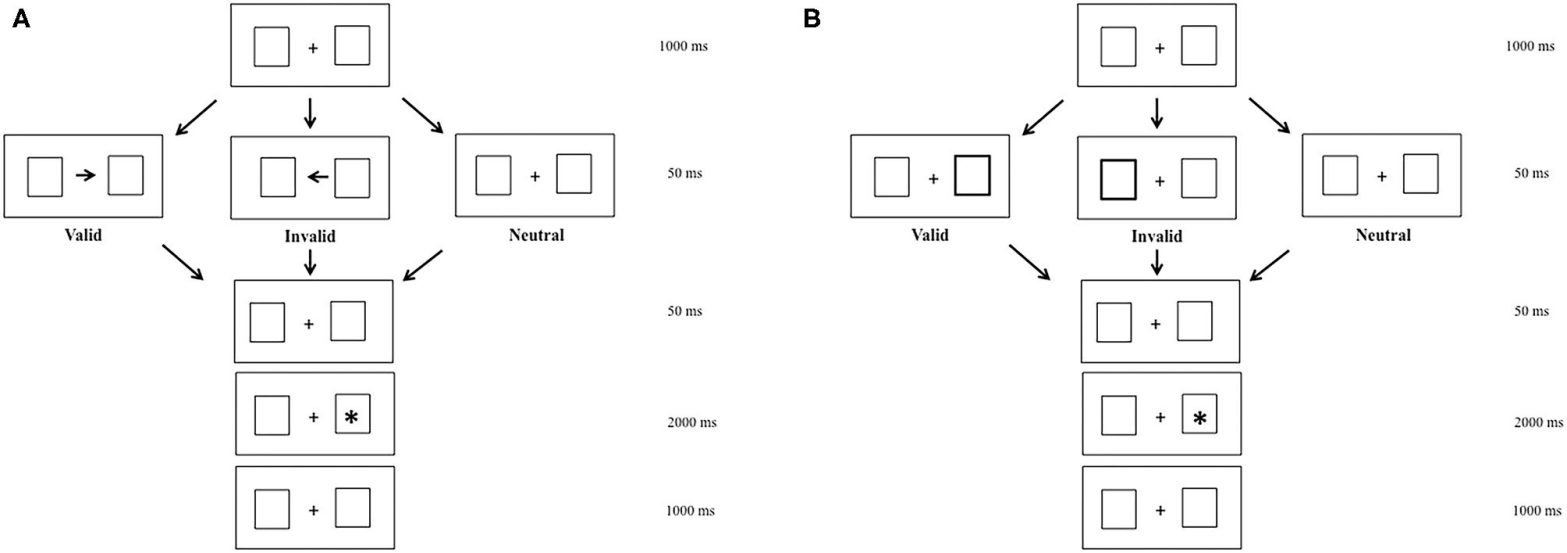
Endogenous **(A)** and exogenous **(B)** modified Posner's conditions in valid, invalid, and neutral sub-conditions.

In the two attention conditions (endogenous vs. exogenous), three visual sub-conditions were presented to participants. The *valid sub-condition* in which arrows and peripheral luminance indicated the location of target, the *neutral sub-condition* in which there were no arrows and peripheral luminance, and the *invalid sub-condition* in which arrows and peripheral luminance indicated the opposite location of the target (Figure 1). For the endogenous and exogenous conditions, there was a fixation period of 1,000 ms, during which children had to fixate a central cross. After that, depending on the valid or invalid sub-conditions, an arrow or peripheral luminance appeared for 50 ms. In the neutral sub-condition, there were not arrows or peripheral luminance, but only the central cross. Later, the central cross reappeared during 50 ms. After that, a target appeared during 2,000 ms.

All children were instructed to look at the target as accurately and as rapidly as possible. Each child performed the two blocks (endogenous and exogenous conditions) of eye movements. Each block was separated by a few minutes of rest. Each block contained 41 random trials: 16 in the valid sub-condition, 9 in the neutral sub-condition, and 16 in the invalid sub-condition. Note that in the original Posner task, the number of trials is more important, particularly that of the valid sub-condition, and the response was manual (24). In the literature, the original Posner task has been adapted, and in the present study the small number of trials was decided because of the age and pathologies of children, tested also because of eye movement's recording. Indeed, recent paper (25) recording eye movements in children of about 9 years old used a number of trials similar to ours. Note that the results of eye movements' latency had a similar order of magnitude.

### Eye Movement Recording

Fixation performance was recorded by the Mobile EBT Tracker (www.SuriCog.fr), a CE-marked medical eye-tracking device. The Mobile EBT is equipped with cameras that capture the movements of each eye independently. The frequency of recording was set up to 300 Hz, and system precision was 0.25°. There was no obstruction of the visual field with this recording system. Children were seated in a chair in a dark room, with the head stabilized by a forehead and chin support; viewing was binocular, and the viewing distance was 60 cm.

Calibration factors for each eye were determined from the eye positions during the calibration procedure (26). Eye movement analyses were performed using the MeyeAnalysis software, which automatically determined the onset and the end of each saccade by using a built-in saccade detection algorithm. A control of the coherence of the analysis was performed by the investigator. For each saccade recorded in the different conditions (endogenous vs. exogenous), we examined their latency in ms, i.e., the time between the onset of the target and the beginning of the eye movements. We recorded also the mean error rate: an error consisted to look at the opposite direction of the target.

### Statistical Analysis

As Shapiro–Wilk test demonstrated that the data were not normally distributed, and all statistics were non-parametric. The non-parametric Mann–Whitney *U*-test was used to compare the latency values and the number of errors made by the two groups of children tested (ADHD and TD children) in the two attention conditions (endogenous and exogenous condition) for the three visual sub-conditions (valid, neutral, and invalid). In order to evaluate the strength of a statistical claim, we calculated the effect size for all variables (*r* = Z/√N) (27). Note that the interpretation of values of effect size is 0.10– <0.30 small, 0.30–0.50 medium, and ≥0.50 large (28). In order to make multiple-comparison 2 attention conditions × 3 visual sub-conditions, we used the Wilcoxon test. When necessary, Bonferroni *post-hoc* comparisons were employed. Analyses were performed using the Statistica software, the GLM (advanced linear models) software, and the level of significance was kept at 0.05.

## Results

**Figure 2:**
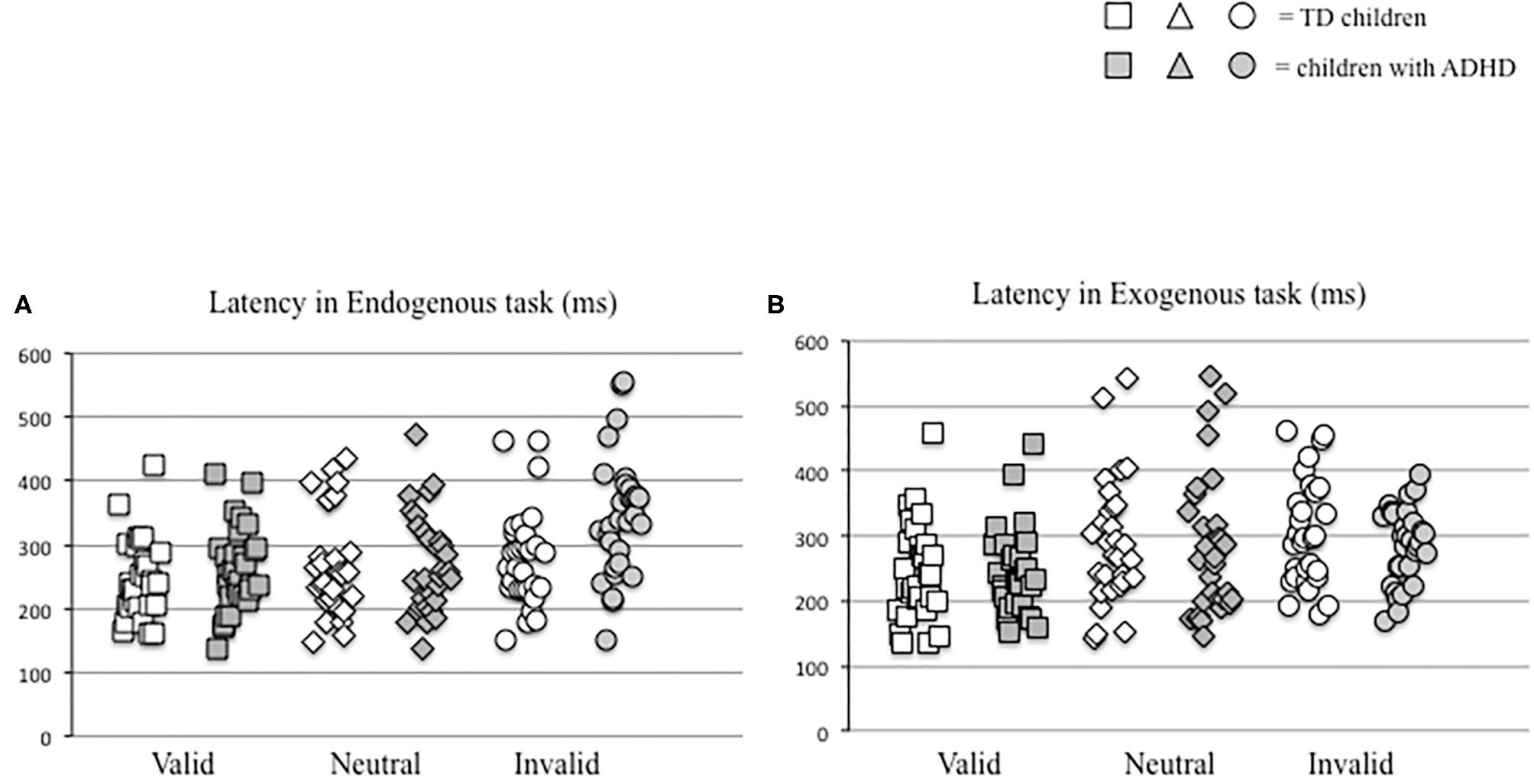
Individual eye movement latency values made by each child in endogenous **(A)** and exogenous **(B)** conditions in the three different visual sub-conditions (valid, neutral, and invalid) considered.

The latency values made by each child tested in the two distinct attention conditions (endogenous vs. exogenous) at the three visual sub-conditions (valid, neutral, and invalid) were reported in Figure 2. The Mann–Whitney *U*-test (see Table 2A) showed a significant difference of latency in the endogenous condition for the invalid sub-condition (*p* < 0.001); children with ADHD showed longer values of latency in the endogenous condition for the invalid sub-condition, when compared to TD children.

**Table 2:** Non-parametric Mann–Whitney U results. Bold values for latency (A) and number of errors (B).

**Latency (ms)**	**Rank sum (TD)**	**Rank sum (ADHD)**	***U***	***Z***	***P*-level**	**Effect size**
**(A)**						
Endogenous—valid	859	1,032	394	−1.02	0.31	−0.13
Endogenous—neutral	875	954	440	−0.13	0.89	−0.01
Endogenous—invalid	715	1,176	250	−3.10	**0.001**	−0.39
Exogenous—valid	935	953	457	0.11	0.91	0.01
Exogenous—neutral	983	728	322	1.52	0.12	0.19
Exogenous—invalid	970	921	425	0.58	0.56	0.07
**Number of error**	**Rank sum (TD)**	**Rank sum (ADHD)**	***U***	***Z***	***P*****-level**	**Effect size**
**(B)**						
Endogenous—valid	811	1,079	346	−1.71	0.06	−0.21
Endogenous—neutral	776	1,054	341	−1.60	**0.03**	−0.20
Endogenous—invalid	811	1,080	346	−1.72	0.07	−0.22
Exogenous—valid	875	1,015	410	−0.79	0.38	−0.10
Exogenous—neutral	724	1,106	289	−2.37	**0.004**	−0.30
Exogenous—invalid	764	1,127	299	−2.39	**0.01**	−0.30

**Figure 3:**
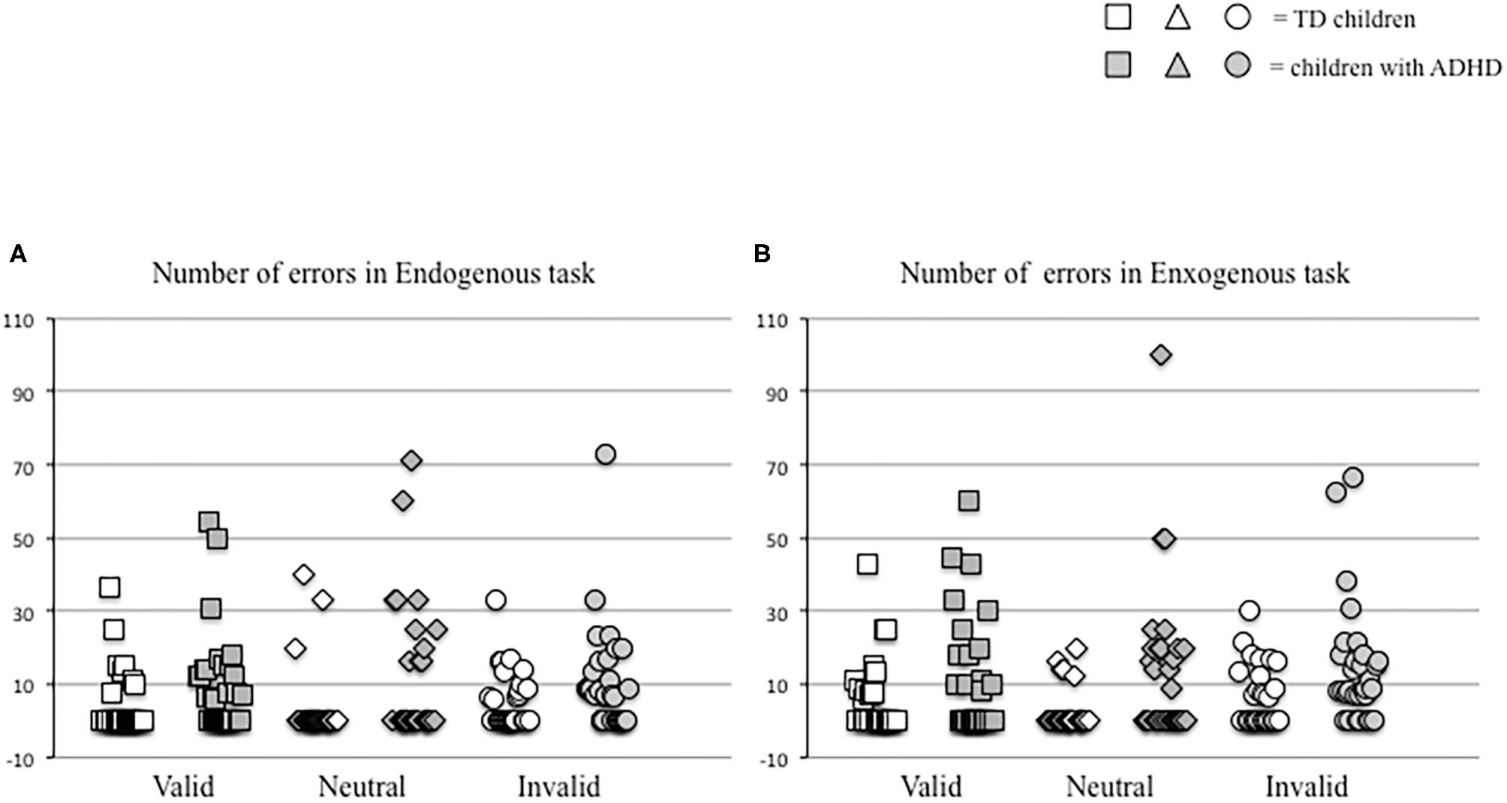
Individual values of number of errors made by each child in endogenous **(A)** and exogenous **(B)** conditions in the three different visual sub-conditions (valid, neutral, and invalid) tested.

In parallel, we measured the number of errors made by each child in the two distinct attention conditions (endogenous vs. exogenous) at the three visual sub-conditions (valid, neutral, and invalid) (Figure 3). The Mann–Whitney *U*-test (see Table 2B) showed a significant difference of the number of errors in the endogenous condition for the neutral sub-condition (*p* < 0.03), and in the exogenous condition for the neutral and invalid sub-condition (*p* < 0.004 and *p* < 0.01, respectively); children with ADHD showed a higher number of errors in the endogenous condition for the neutral sub-condition and in the exogenous condition for the neutral and invalid sub-condition when compared to TD children.

The Wilcoxon test (see Tables 3A,B) showed a significant difference concerning the values of latency in the invalid sub-condition only in children with ADHD.

**Table 3:** Non-parametric Wilcoxon results for latency (A) and number of errors (B).

	**TD children**			**Children with ADHD**				
**Latency (ms)**	***T***	***Z***	***P*-level**	***T***	***Z***	***P*-level**	**Bonferroni *post-hoc***	**Effect size**
**(A)**								
Endogenous—valid vs. exogenous valid	859	1,032	394	−1.02	0.31	−0.13		
Endogenous—neutral vs. exogenous neutral	875	954	440	−0.13	0.89	−0.01		
Endogenous—invalid vs. exogenous invalid	715	1,176	250	−3.10	**0.001**	−0.39	0.003	0.57
**Errors**	***T***	***Z***	***P*****-level**	***T***	***Z***	***P*****-level**	**Bonferroni** ***post-hoc***	**Effect size**
**(B)**								
Endogenous—valid vs. exogenous valid	44	0.9	0.36	128	0.30	0.76		
Endogenous—neutral vs. exogenous neutral	10.50	1.05	0.29	110	0.17	0.86		
Endogenous—invalid vs. exogenous invalid	57.50	1.21	0.22	114	1.29	0.19		

## Discussion

The aim of the present study was to explore attention shift impairment in ADHD. We examined the eye movement latency in an adjusted Posner's task in children with ADHD compared to age-, sex-, and IQ-matched typically developing children. Our results were in accordance with our initial hypothesis. Children with ADHD showed longer latency during the endogenous task for the invalid sub-condition and made more errors than did TD children errors in the endogenous condition for the neutral sub-condition and in the exogenous condition for the neutral and invalid sub-condition.

### Endogenous and Exogenous Tasks: Difference Between Typically Developing Children vs. Children With ADHD Concerning Values of Latency

The modified Posner's task that we used in our study is a neuropsychological test in which two major types of cues are commonly applied to analyze attention shift based on the distinct visual inputs. The task paradigm relies on the hypothesis that exogenous attention is more driven by peripheral inputs and endogenous attention by central inputs: peripheral signals directly indicate the location of the target and solicit reflective attention, whereas the central signals, before being used, need be interpreted cognitively in order to voluntarily engage attention (29). Previous findings in the literature supported that attention shift related to both sources of tasks (endogenous vs. exogenous) did not share the same cerebral networks (30).

Attention shift related to exogenous stimuli recruited a dorsal frontoparietal network, particularly the dorsal parietal cortex (intraparietal sulcus and superior parietal lobule) and the dorsal frontal cortex along the precentral sulcus, near or in the frontal eye field. In contrast, attention shift related to endogenous tasks included not only the dorsal but also the ventral fronto-parietal network, which included the temporoparietal junction cortex (the gyrus and the ventral part of the supra-marginal gyrus) and the ventral frontal cortex (including parts of the middle frontal gyrus, the inferior frontal gyrus, the frontal operculum, and the anterior insula) (3).

In the present study, in which, for the first time, eye-tracking was used to record eye movement latency in children with ADHD, we reported significant longer latency for children with ADHD only in the endogenous condition for the invalid sub-condition when compared to TD children. Our result reinforced previous findings, suggesting that children with ADHD have difficulties to voluntarily disengage their attention from an erroneous condition (invalid condition) in order to engage attention in the correct direction (31, 32). Recently, in an fMRI study exploring the Posner task performance in 11 healthy adults, Meyer et al. (33) showed that during the endogenous condition and invalid sub-condition, the cerebellar frontal activity of the left hemisphere was greater with respect to valid sub-condition. In line with this article, we could suggest that children with ADHD may have a deficiency in cortical frontal networks.

### Endogenous and Exogenous Tasks: Difference Between Typically Developing Children vs. Children With ADHD Concerning Number of Errors

In our study, results showed that children with ADHD have a tendency to make more frequent errors in both conditions than children with TD even if the number of errors was significant for the endogenous condition in the neutral sub-condition and for the exogenous condition in both neutral and invalid sub-conditions (see Table 2B). These findings may be explained by a defect of inhibitory control in subjects with ADHD. Previous findings reported consistently an inhibitory deficit in executive processes in ADHD (34) associated with voluntary saccade abnormalities (35) leading to an elevated number of errors for the two neutral sub-conditions (endogenous as well as exogenous conditions). The dorso-lateral prefrontal cortex and frontal eye field have both a major role for saccade inhibition (36) but also for inhibitory activity (37, 38). This suggests a presence of a dysfunction of the dorso-lateral prefrontal cortex in children with ADHD even if further neuroimaging studies will be necessary to confirm this hypothesis.

Finally, we could develop other types of experiments for exploring attention on such clinical population by comparing social stimuli (such as faces) vs. neutral stimuli (such as arrows) as done by Boncompagni and Casagrande (39), given that positive or negative emotion influenced response from the attentional network.

## Limitations

Further studies with a larger number of children with ADHD with different types of comorbidities will be necessary in order to evaluate more accurately the attentional skills in this type of pathology. The low number of trials could be considered a limitation of this study; however, this could be explained by the fact that we did not want to tire children, particularly children with ADHD when latency of eye movements was recorded. Note also that in the literature studies leading with the Posner task in children, a shorter version of the Posner task was used even when the manual (40) or latency (25) response was recorded.

## Conclusion

Overall, our study suggested that children with ADHD may allocate their attention resource toward the detection of exogenous targets with a deficit in their ability to perform an endogenous task. This could result from impaired interactions between the ventral and dorsal attention networks as well as in the frontal eye field. However, we observed that children with ADHD were able to use different type of attention indicators (alerting, orienting, and reorienting) like typically developing children.

In the future, it could be interesting to combine behavioral and neuroimaging studies for better understanding the neurophysiological hypotheses of the attentional deficits in subjects with ADHD.

## Data Availability Statement

All datasets presented in this study are included in the article/supplementary material.

## Ethics Statement

The studies involving human participants were reviewed and approved by Comité de Protection des Personnes CPP, Ile de France. Written informed consent to participate in this study was provided by the participants' legal guardian/next of kin.

## Author Contributions

MB, SC, and FI: conceptualization. MS and EA: selection of patients. SC and MB: oculomotor measure and data analysis. SC and MB: writing original draft. SC, MB, RD, and FI: review and editing. All authors: contributed to the article and approved the submitted version.

## Conflict of Interest

The authors declare that the research was conducted in the absence of any commercial or financial relationships that could be construed as a potential conflict of interest.
